# First person – Sarah Colijn

**DOI:** 10.1242/dmm.044024

**Published:** 2020-01-24

## Abstract

First Person is a series of interviews with the first authors of a selection of papers published in Disease Models & Mechanisms (DMM), helping early-career researchers promote themselves alongside their papers. Sarah Colijn is first author on ‘Cell-specific and athero-protective roles for RIPK3 in a murine model of atherosclerosis’, published in DMM. Sarah conducted the research described in this article while a graduate student in Courtney Griffin's lab at Oklahoma Medical Research Foundation, Oklahoma City, OK, USA. She is now a Postdoctoral Research Associate in the lab of Amber Stratman at Washington University in St. Louis, School of Medicine, St. Louis, MO, USA, investigating how the cardiovascular system develops during embryogenesis in both physiological and pathological scenarios.


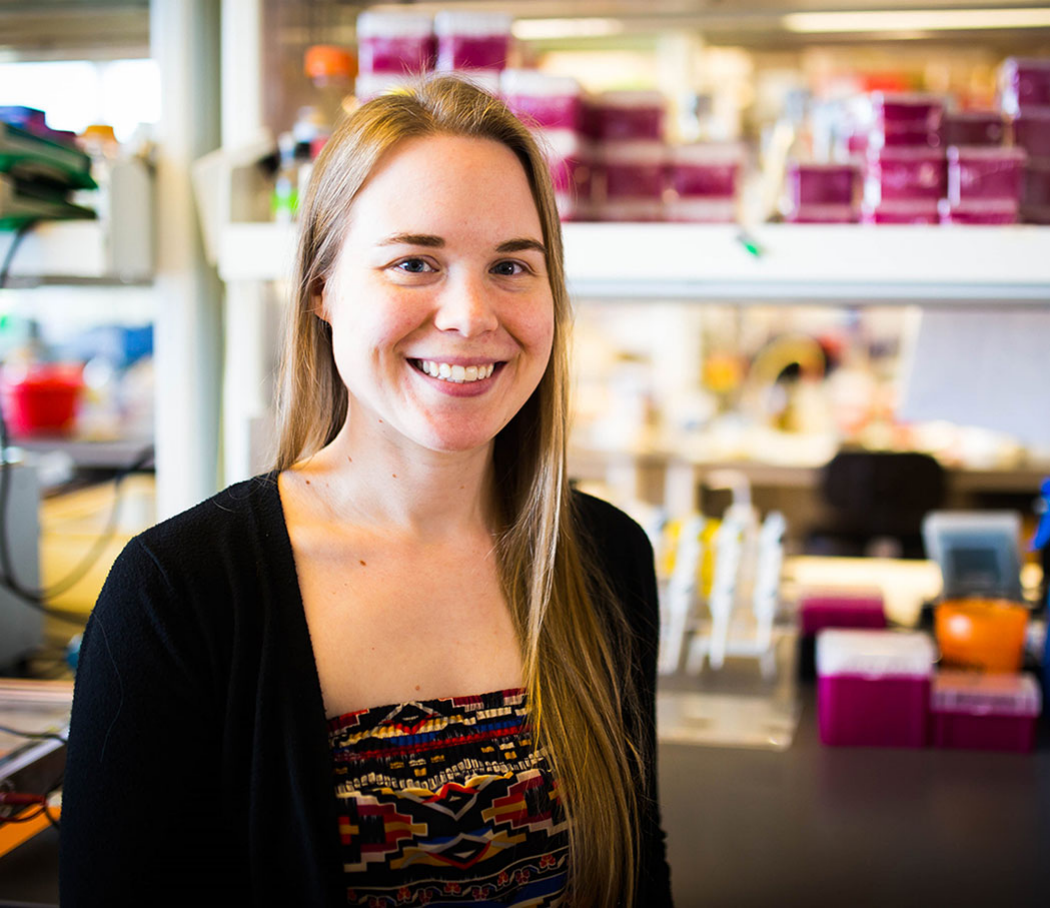


**Sarah Colijn**

**How would you explain the main findings of your paper to non-scientific family and friends?**

Atherosclerosis is so common that nearly everyone has it, yet very few people seem to know what it actually is. It is commonly referred to as the ‘hardening of the arteries’ and is characterized by the buildup of cholesterol, lipids, dead cells and other debris in the blood vessel wall. Despite the fact that atherosclerosis is the leading cause of heart disease and is one of the most highly studied diseases, there are few drug treatments that provide satisfactory results. Recently, researchers have proposed that one druggable target to prevent atherosclerosis could be the cell death pathway known as necroptosis. Necroptosis is considered to be harmful because it can cause inflammation and can theoretically make atherosclerosis worse. However, we have found that inhibiting a certain protein in the necroptosis pathway, RIPK3, could have unexpected and detrimental effects on atherosclerosis progression. In fact, RIPK3 protects against atherosclerosis in certain cell types, making it a poor target for drug design.

**What are the potential implications of these results for your field of research?**

This work challenges the atherosclerosis and necroptosis fields to rethink the role of RIPK3 in certain contexts and to acknowledge that it can function in a non-pathological way. Previously, necroptosis was considered to be inherently inflammatory and overall detrimental in any disease context. However, the function of one of the essential components of necroptosis, the kinase RIPK3, is proving to be more complex than we previously thought. In fact, we suspect that RIPK3 functions in a multitude of pathways that have very little to do with necroptosis. This is becoming clearer as we study RIPK3 in the vasculature during embryogenesis and postnatally.

“This work challenges the atherosclerosis and necroptosis fields to rethink the role of RIPK3 in certain contexts.”

**What are the main advantages and drawbacks of the model system you have used as it relates to the disease you are investigating?**

We use the well-established *Apoe^−/−^* mouse line as our animal model to study atherosclerosis. The atherosclerotic plaques that develop in these mice share many similarities with human plaques in terms of atherosclerosis initiation, progression and inflammation. Mice are also relatively easy to genetically manipulate, which allows us to use conditional knockout approaches to study our protein of interest. However, the *Apoe^−/−^* mouse model does not often experience ‘plaque rupture’, which is the deadliest aspect of atherosclerosis in humans. Therefore, although the model mimics most aspects of the progression of human atherosclerosis, it is not the right model for studying end-stage heart disease and plaque rupture.

**What has surprised you the most while conducting your research?**

Based on previous reports, we expected that cell-specific deletion of RIPK3 in macrophages would protect against atherosclerosis. In reality, macrophage- and endothelial cell-specific deletion of RIPK3 worsened the disease. This came as a complete surprise because the cell death literature had already established RIPK3 as a harmful protein. Our studies reveal that there is certainly more to how RIPK3 functions in atherosclerosis than meets the eye.

**Figure:**
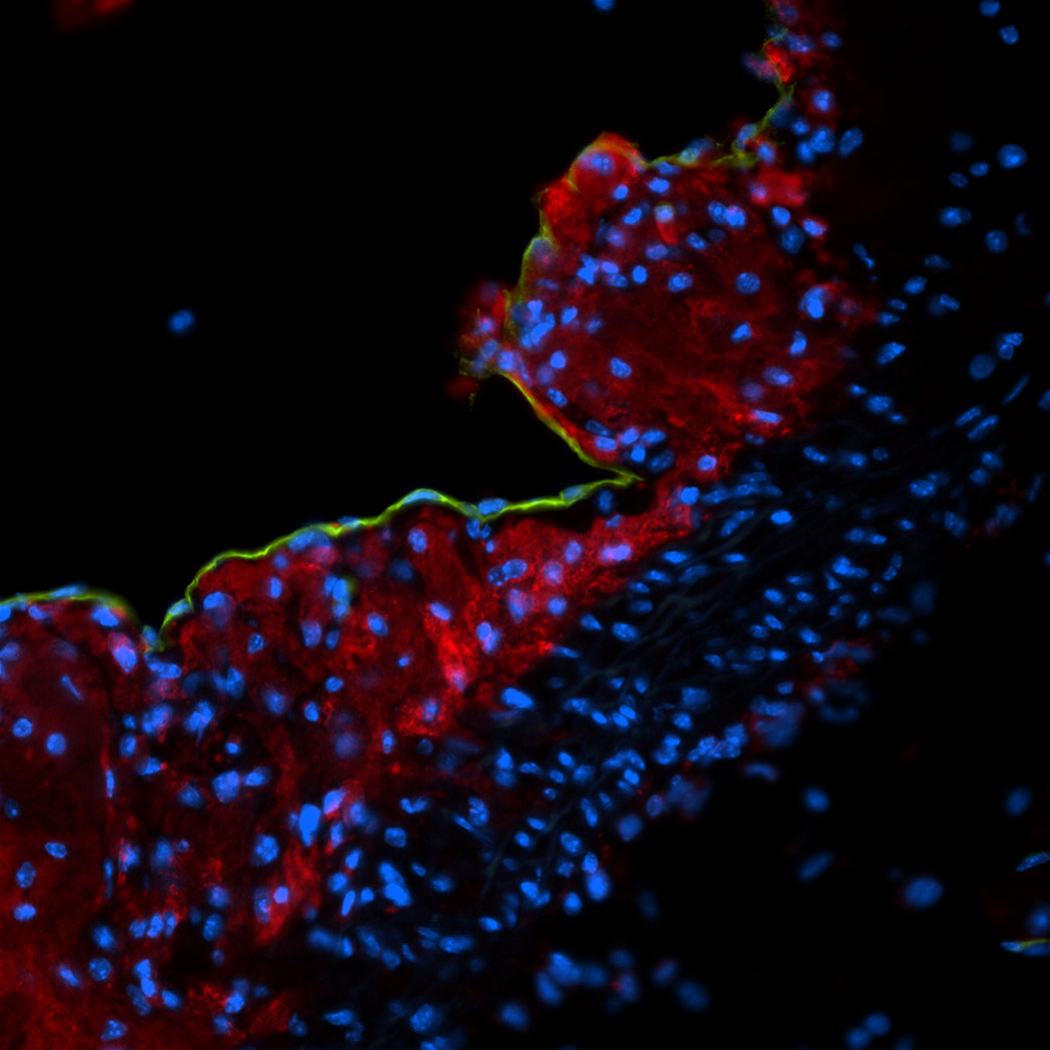
**Atherosclerotic plaque in the aortic root demonstrating immense macrophage infiltration.** Macrophages (CD68; red), endothelial cells (CD31; green) and nuclei (Hoechst; blue).

**What changes do you think could improve the professional lives of early-career scientists?**

I think early-career scientists are the most successful when they have a respectful mentor-mentee relationship. I am very lucky to have had mentors with reasonable expectations and healthy work-life balances. I also feel that I have always been treated more like a colleague than an underling. Too many times I have seen the abuse of the mentor-mentee relationship and have seen the mental and physical toll it can take on the graduate student or postdoc. I think nothing harms a career more than not having that personal advocate early on. There should be more accountability for mentors who take on trainees to make sure that the trainees are treated with respect and civility.

“I think early-career scientists are the most successful when they have a respectful mentor-mentee relationship.”

**What's next for you?**

I am currently a postdoc at Washington University in St. Louis with Dr Amber Stratman. I have shifted animal models and now work with zebrafish to uncover blood-flow-dependent mechanisms that regulate vascular development. I expect that learning this new animal model will increase my technical expertise and allow me to critically decide which animal model would be the best for addressing certain scientific questions.
